# Clinical Determinants Differentiating the Severity of SARS-CoV-2 Infection in Cancer Patients: Hospital Care or Home Recovery

**DOI:** 10.3389/fmed.2021.604221

**Published:** 2021-02-16

**Authors:** Dong D. Lin, Yunhong Wu, Sudhamshi Toom, Niki Sheth, Kevin Becker, Susan Burdette-Radoux, James D'Silva, Yiwu Huang, Jay Lipshitz, Trishala Meghal, Lan Mo, Pooja Murthy, Philip Rubin, Vijaya Natarajan, Bernadine Donahue, Yiqing Xu

**Affiliations:** ^1^Department of Volunteers and Student Services, Maimonides Medical Center, Brooklyn, NY, United States; ^2^Department of Biostatistics, Harvard T.H. Chan School of Public Health, Boston, MA, United States; ^3^Department of Medicine, Division of Hematology/Oncology, Maimonides Medical Center, Brooklyn, NY, United States; ^4^Department of Radiation Oncology, James J. Peters Veterans Affairs Medical Center, Bronx, NY, United States; ^5^Department of Radiation Oncology, Maimonides Medical Center, Brooklyn, NY, United States

**Keywords:** COVID-19, cancer, chemotherapy, immunosuppression, asymptomatic, treatment of negative impact

## Abstract

**Background:** Cancer patients may carry a worse prognosis with SARS-CoV-2 infection. Most of the previous studies described the outcomes of hospitalized cancer patients. We aimed to study the clinical factors differentiating patients requiring hospital care vs. home recovery, and the trajectory of their anti-cancer treatment.

**Methods:** This study was conducted in a community cancer center in New York City. Eligible patients were those who had cancer history and were diagnosed of SARS-CoV-2 infection between March 1 and May 30, 2020, with confirmatory SARs-CoV-2 virus test or antibody test. Four groups were constructed: (A) hospitalized and survived, (B) hospitalized requiring intubation and/or deceased, (C) non-hospitalized, asymptomatic, with suspicious CT image findings, close exposure, or positive antibody test, and (D) non-hospitalized and symptomatic.

**Results:** One hundred and six patients were included in the analysis. Thirty-five patients (33.0%) required hospitalization and 13 (12.3%) died. Thirty (28.3%) patients were asymptomatic and 41 (38.7%) were symptomatic and recovered at home. Comparing to patients who recovered at home, hospitalized patients were composed of older patients (median age 71 vs. 63 years old, *p* = 0.000299), more who received negative impact treatment (62.9 vs. 32.4%, *p* = 0.0036) that mostly represented myelosuppressive chemotherapy (45.7 vs. 23.9%, *p* = 0.0275), and more patients with poorer baseline performance status (PS ≥ 2 25.7 vs. 2.8%, *p* = 0.0007). Hypoxemia (35% in group A vs. 73.3% in group B, *p* = 0.0271) at presentation was significant to predict mortality in hospitalized patients. The median cumulative hospital stay for discharged patients was 16 days (range 5–60). The median duration of persistent positivity of SARS-CoV-2 RNA was 28 days (range 10–86). About 52.9% of patients who survived hospitalization and required anti-cancer treatment reinitiated therapy. Ninety-two percent of the asymptomatic patients and 51.7% of the symptomatic patients who recovered at home continued treatment on schedule and almost all reinitiated treatment after recovery.

**Conclusions:** Cancer patients may have a more severe status of SARS-CoV-2 infection after receiving myelosuppressive chemotherapy. Avoidance should be considered in older patients with poor performance status. More than two thirds of patients exhibit minimal to moderate symptoms, and many of them can continue or restart their anti-cancer treatment upon recovery.

## Introduction

The unprecedented COVID-19 pandemic has presented a public health challenge globally. As of May 31, 2020, 1,778,515 confirmed cases and 104,051 deaths were reported in the US (1). Patients with advanced age and comorbidities appeared to have poorer outcomes with the SARS-CoV-2 infection (2, 3). Cancer patients, as a group, also showed higher fatality rates (2–5). Presumably, factors such as the presence or absence of disease, recent therapy with possible myelosuppressive or immunosuppressive potentials, and types of cancer may play important roles in influencing their outcomes. Most of the previous studies concentrated on the examination of patients who developed severe symptoms and required hospitalization.

In this registry study, we analyzed clinical factors including presence of disease, cancer-related treatment, interval between cancer treatments to clinical diagnosis of SARS-CoV-2 infection or admission, baseline performance status, and immune status of all patients, and compared these frequencies between hospitalized patients and non-hospitalized patients. We hypothesized that anti-cancer treatments with potential negative impact to the immune system and the interval between its administration and the onset of COVID-19 symptoms may be critical. We defined this category of therapy to include myelosuppressive, immunosuppressive, and immune modulating agents. We also evaluated time kinetics of virus clearance and time kinetics of re-initiation of anti-cancer treatment. We further followed the trajectory of recovery of the patients and studied the time course of persistent positivity of SARS-CoV-2 RNA.

## Methods

This was a prospective observational registry study established in March 2020 and approved by Institutional Review Board (IRB). Patients were eligible if they had a confirmed or suspicious diagnosis of SARS-CoV-2 infection between March 1, 2020 and June 15, 2020, as well as active cancer history. Two cohorts of patients were enrolled. In cohort 1, patients were identified by health care providers between March and May of 2020 when they presented with suspicious COVID-19 symptoms, or had a known close contact to a known COVID-19 case, or suspicious radiological findings on CT or X-rays performed for the purpose of their cancer staging. A suspicious radiographic reading was defined as “peripheral ground-grass opacities,” possibly compatible with SARS-CoV-2 infection. As COVID-19 reverse transcriptase-polymerase chain reaction (RT-PCR) tests were only offered to patients who met the hospitalization criteria (usually hypoxemia with oxygen saturation <92% at room air), some patients did not get RT-PCR test at the time of suspicion, and were then followed up and offered COVID-19 antibody tests when it became available starting May 2020. Only those who had confirmed SARS-CoV-2 diagnosis, defined by positive COVID-19 RT-PCR or positive COVID-19 antibody test were included in the main analysis. One exception was a patient who died quickly in the hospital and did not have an opportunity for testing but carried highest clinical suspicion. In cohort 2, patients were retrospectively identified through electronic medical records by their positive COVID-19 RT-PCR test, positive point of care virus test, or positive COVID-19 antibody test, which were performed between May 20 and June 30, 2020. Medical records were reviewed for documentation of symptoms, treatment history, laboratory and radiological findings, and admission records. For the patients in cohort 2, if the COVID-19 related symptoms were not documented in the medical records, health care providers conducted interviews by phone-calls to help patients to recall their symptoms potentially associated with their past SARS-CoV-2 infection, and to get contact history.

Nasopharyngeal swabs were collected and tested for SARS-CoV-2 RNA with RT-PCR assay using the XPERT XPRESS SARS-COV-2 test kit in our hospital laboratory. Nasopharyngeal swabs were also collected for the molecular point-of-care test for SARS-CoV-2 virus detection using Abbott ID NOW™ kit. Antibody tests (IgG and IgM) were sent out and tested at Lenco Diagnostics Laboratory, Brooklyn, NY. The data entry cut off was 7/1/2020.

### Study Group Definition

Patients in cohort 1 and 2 were combined and divided into 4 groups. Group A patients were hospitalized with no intubation events, discharged, and survived. Group B patients were hospitalized and required intubation or hospitalized and were deceased. Group C patients were asymptomatic who were tested for a suspicious CT scan result, had a history of close exposure to a known case, or did not recall any symptoms after showing presence of positive COVID-19 Ig G or Ig M antibodies. Group D patients exhibited symptoms consistent with SARS-CoV-2 infection, though these symptoms were not severe enough for hospital admission.

### Definition of Performance Status and Baseline Immune Status

The performance status scale (PS) was based on Eastern Cooperative Oncology Group (ECOG) scale. PS 0: fully active, no performance restrictions; PS1: restricted in strenuous physical activity, fully ambulatory and able to carry out light work; PS2: Capable of all self-care but unable to carry out any work activities, up and about >50% of waking hours; PS3: Capable of only limited self-care, confined to bed or chair >50% of the waking hours. PS4: Completely disabled, cannot carry out any self-care; totally confined to bed or chair (6).

The baseline immune status was estimated by taking lab results performed in February 2020 or prior, including absolute neutrophil count, absolute lymphocyte count, and albumin level. Any abnormal value among the three tests, defined as lower than the lower limit of the normal range, was considered abnormal.

### Treatment Category Definition

Myelosuppressive regimens included all routine chemotherapy drugs. Exceptions include therapeutic antibodies (trastuzumab, bevacizumab), oral targeted therapies (erlotinib, osimertinib), and hormonal treatments (luteinizing hormone releasing hormone (LHRH) agonists, fulvestrant, tamoxifen, and aromatase inhibitors). Immunosuppressive drugs included rituximab, lenalidomide, high dose steroids, and daratumumab. Immune modulating agents included the immune checkpoint inhibitors that target PD-1 (programmed cell death protein 1) or PD-L1, such as pembrolizumab, nivolumab, and durvalumab. Negative impact treatment denotes treatments with potential negative impact on the immune system (any regimens in the myelosuppressive, immunosuppressive, or immune modulating categories).

### Definition of Treatment Duration

The start of any anti-cancer treatment until the day of diagnosis of SARS-CoV-2 infection. If there was a treatment break of more than 3 months, then the treatment before the break was not counted. If the continuation of treatment included hormonal or non-myelosuppressive treatment followed by treatment with negative impact, then the start day of treatment with negative impact treatment was chosen for the start day.

### Sampling Frequency Study of the Time Kinetics of Persistent SARS-CoV-2 Virus Status

Patients were screened for whether or not a repeat COVID-19 RT-PCR test were performed. Most of those tests were done at unplanned intervals mainly to get a negative result to qualify patients to resume anti-cancer treatment. The days between the initial positive test and the last positive test was defined as “positive duration.”

### Statistical Analysis

We first compared the clinical features of hospitalized patients (Group A vs. B). Next, we compared the clinical features of the hospitalized patients to at-home patients (Group A + B vs. Group C + D). Wilcoxon rank sum test or Student's *t*-test were used to compare continuous data. Fisher's exact test was performed for the categorical variables. Next, we assessed risk factors for hospitalization—a more severe status of SARS-CoV-2 infection. We conducted multivariate analyses by utilizing logistic regression with the inclusion of variables significant in univariate analysis. The multivariate logistic model was built from a two-sided stepwise regression based on the Akaike Information Criterion (AIC). AIC is an estimator of out-of-sample prediction error and thereby relative quality of statistical models for a given set of data. Given a collection of models for the data, AIC estimates the quality of each model, relative to each of the other models. Thus, AIC provides a means for model selection.

Odds ratios for a more severe status of SARS-CoV-2 infection were calculated via logistic regression. Two-sided significance level 0.05 was used. All statistical analyses were done using R (version 3.5.3; The R Foundation).

## Results

### Patient Demographics

**Figure 1:**
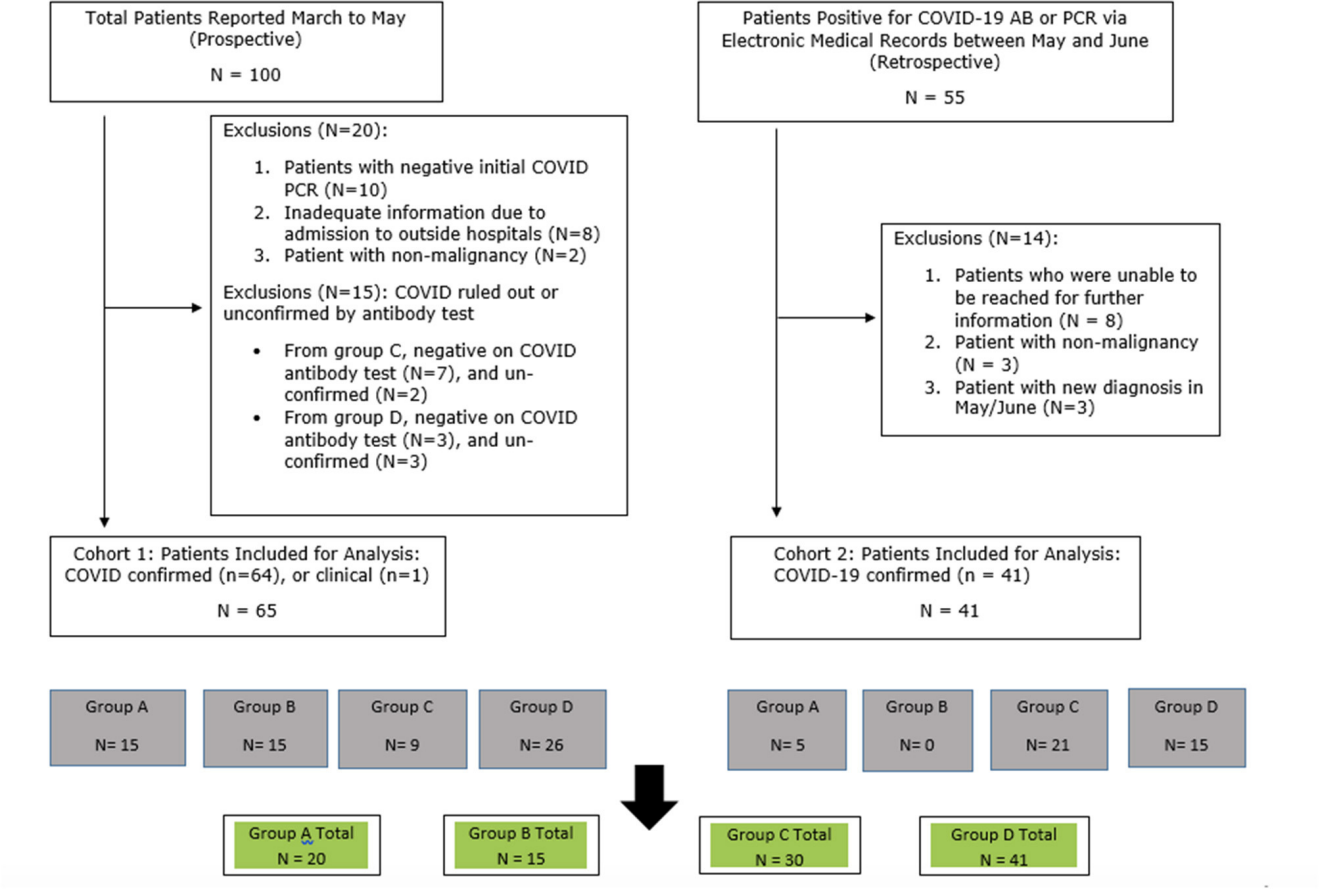
Flow chart of patient selection.

Out of the 100 cases with clinical suspicious diagnosis of SARS-CoV-2 infection which were reported to the study team between March and May 2020, 65 patients were included in cohort 1 of this analysis (Figure 1). Out of the 55 patients identified retrospectively by electronic medical records, 41 were included in the analysis (Figure 1). For all of the 106 eligible patients, the median age was 65 years old (range 31–94). There was female predominance (69.8%), and 44.3% were Caucasian (Table 1). The female predominance was present in all 4 groups and was numerically highest in group D (Table 2). Ninety-five (89.6%) patients had solid tumors, with the most common being breast cancer (36.8% of total). Forty-seven (44.3%) patients had no evidence of disease (NED). Fifty-nine patients (55.7%) had presence of tumor, either localized (17.9%) or metastatic (37.7%). Seventy-eight (73.6%) patients were receiving active treatment within 3 months of diagnosis, and 45 (42.5%) were receiving negative impact treatment. Thirty-six (34.0%) who were receiving negative impact treatment received last dose of therapy within 30 days of SARS-CoV-2 diagnosis. Thirty-five (33.0%) patients were hospitalized and the remainder recovered at home (Table 1). The detailed breakdowns of the demographic and clinical characteristics in the four groups are listed in Table 2.

**Table 1:** Patient demographics and disease characteristics.

**Characteristics**	**Total number (%)**
Total number	106
Age	
Median	65
Range	31–94
Gender	
Male	32 (30.2)
Female	74 (69.8)
Ethnicity	
Caucasian	47 (44.3)
African American	32 (30.2)
Asian	10 (9.4)
Hispanic	15 (14.2)
Mid-Eastern	2 (1.9)
Cancer types	
**Solid tumors**	**95 (89.6)**
Breast	39 (36.8)
GYN	13 (12.3)
Gastrointestinal	12 (11.3)
Lung	12 (11.3)
Head/Neck	5 (4.7)
GU	12 (11.3)
Brain	1 (1.5)
Osteosarcoma	1 (1.5)
**Hematological malignancy**	**11 (10.4)**
Presence of disease	
**No evidence of disease (NED)**	**47 (44.3)**
**Presence of disease**	**59 (55.7)**
Localized disease	19 (17.9)
Metastatic disease	40 (37.7)
Treatment	
No treatment	28 (26.4)
Active treatment^*^	78 (73.6)
Negative impact treatment	45 (42.5, 45/106)
Treatment within 30 days	36 (34.0, 36/106)
Diagnosis month	
March	22 (20.8)
April	54 (50.9)
May	11 (10.4)
June	1 (0.9)
Unknown	18 (17.0)
Hospitalized	35 (33.0)
Staying home	71 (67.0)

* *Defined as last treatment (any anti-cancer) treatment within 3 months of SARS-CoV-2 diagnosis*.

**Table 2:** Clinical characteristics of patients divided by groups of severity of SARS-CoV-2 infection.

**Characteristics**	**Group A Hospitalized**	**Group B Intubated and/or Deceased**	***p*-Value *A* vs. *B***	**Group C Asymptomatic Suspicious CT**	**Group D Symptomatic, Home Isolation**	**Group A + B**	**Group C + D**	***p*-Value** ** A + B vs.** ** C + D**
Total number	20 (18.9)	15 (14.2)		30 (28.3)	41 (38.7)	35 (33.0)	71 (67.0)	
Confirmed^*^	20 (4 AB^*^)	14		30 (27 AB)	41 (35 AB)	34 (4 AB)	71 (62 AB)	
Unconfirmed	0	1		0	0	1	0	
Age								
Median	74	68	0.4593	68	60	71	63	**0.000299**
Range	44–88	48–83		31–87	43–94	44–88	31–94	
Gender								
Male	8 (40)	6 (40)	1	11 (36.7)	7 (17.1)	14 (40)	18 (25.4)	0.1764
Female	12 (60)	9 (60)		19 (63.3)	34 (82.9)	21 (60)	53 (74.6)	
Cancer types								
Solid tumors	16 (80)	13 (86.7)	0.6804	29 (96.7)	37 (90.2)	29 (82.9)	66 (93)	0.172
Hematological	4 (20)	2 (13.3)		1 (3.3)	4 (9.8)	6 (17.1)	5 (7)	
Disease status								
NED	9 (45.0)	3 (20)	0.1629	16 (53.3)	19 (46.3)	12 (34.3)	35 (49.3)	0.1534
Presence of disease	11 (55.0)	12 (80)		14 (46.7)	22 (53.7)	23 (65.7)	36 (50.7)	
Localized	4 (20)	3 (20)		7 (23.3)	5 (12.2)	7 (20)	12 (16.9)	
Metastatic	7 (35.0)	9 (60)		7 (23.3)	17 (41.5)	16 (45.7)	24 (33.8)	
Treatment and disease								
NED no Tx	4(20)	1 (6.7)	0.511	4 (13.3)	8 (19.5)	5 (14.3)	12 (16.9)	1
NED + Tx	5 (25.0)	2 (13.3)		12 (40)	11 (26.8)	7 (20)	23 (32.4)	0.2522
+ disease no Tx	2 (10.0)	2 (13.3)		3 (10)	4 (9.8)	4 (11.4)	7 (9.9)	1
+ disease + Tx	9 (45.0)	10 (66.7)		11 (36.7)	18 (43.9)	19 (54.3)	29 (40.8)	0.2175
Treatment								
Yes	14 (70.0)	12 (80)	0.7	23 (76.7)	29 (70.7)	26 (74.3)	52 (73.2)	1
No	6 (30)	3 (20)		7 (23.3)	12 (29.3)	9 (25.7)	19 (26.8)	
Treatment								
Myelosuppressive	8 (40.0)	8 (53.3)	0.506	6 (20)	11 (26.8)	16 (45.7)	17 (23.9)	**0.0275**
Immunosuppressive	1 (5.0)	0	1	0	1 (2.4)	1 (2.9)	1 (1.4)	1
Immunomodulating	3 (15)	2 (13.3)	1	3 (10)	2 (4.9)	5 (14.3)	5 (7.0)	0.2926
Any above negative impact	12 (60)	10 (66.7)	0.7372	9 (30)	14 (34.1)	22 (62.9)	23 (32.4)	**0.0036**
Negative Impact <30 days	10 (50)	7 (46.7)	1	8 (26.7)	11 (26.8)	17 (48.6)	19 (26.8)	**0.0171**
Time duration of active treatment (m)								
Median (range)	3.1 (0.6–81.3)	3.0 (0–46.1)	0.2364	9.8 (0.7–115.4)	13.4 (1.6–63.9)	3.0 (0–81.3)	11.2 (0.7–115.4)	**0.0058**
Mean (SD)	14.9 (0.47)	6.5 (0.41)		17.0 (0.43)	19.8 (0.46)	11.0 (0.44)	18.6 (0.45)	
Time duration of tx with negative impact (m)								
Median (range)	2.7 (0.6–33.1)	3.0 (0–46.1)	0.4281	8.5 (0.7–115.4)	6.5 (1.8–48.5)	2.7 (0–46.1)	8.0 (0.7–115.4)	0.0823
Mean (SD)	10.3 (0.50)	7.0 (0.49)		20.0 (0.47)	12.4 (0.48)	8.8 (0.49)	15.4 (0.47)	
Chemotherapy before admission								
Median (days)	7	13.5	0.1112					
Range (days)	1–64	1–79						
Less than 14 Days	9 (45)	6 (40)	1					
Greater than 14 Days	3 (15)	4 (26.7)	0.4301					
No treatment	8 (40)	5 (33.3)	0.7372					
Hypoxemia on presentation to the hospital								
Yes	7 (35)	11 (73.3)	**0.0271**					
No	6 (30)	0						
Unknown	7 (35)	4 (26.7)						
Comorbidities								
0 or 1 factor	7 (35)	2 (13.3)	0.2444	16 (53.3)	21 (51.2)	9 (25.7)	37 (52.1)	**0.0124**
2 or more factors	13 (65)	13 (86.7)		14 (46.7)	20 (48.8)	26 (74.3)	34 (47.9)	
HTN	13 (65)	14 (93.3)	0.1009	18 (60)	24 (58.5)	27 (77.1)	42 (59.2)	0.0844
DM	6 (30)	7 (46.7)	0.481	8 (26.7)	13 (31.7)	13 (37.1)	21 (29.6)	0.5085
HLD	9 (45)	8 (53.3)	0.738	11 (36.7)	8 (19.5)	17 (48.6)	19 (26.8)	**0.0309**
History of PE/DVT	4 (20)	1 (6.7)	0.365	1 (3.3)	3 (7.3)	5 (14.3)	4 (5.6)	0.1526
COPD/ILD/asthma	4 (20)	2 (13.3)	0.6804	6 (20)	5 (12.2)	6 (17.1)	11 (15.5)	1
Baseline lab for immune status								
Abnormal	8 (40)	3 (20)	0.2814	7 (23.3)	7 (17.1)	11 (31.4)	14 (19.7)	0.2254
Normal	12 (60)	11 (73.3)	0.4885	23 (76.7)	29 (70.7)	23 (65.7)	52 (73.2)	0.4975
Unknown	0	1 (6.7)	0.4286	0	5 (12.2)	1 (2.9)	5 (7)	0.6611
Performance status (PS)								
PS 0–1	13 (65)	11 (73.3)	0.721	29 (96.7)	36 (87.8)	24 (68.6)	65 (91.5)	**0.0042**
PS ≥2	6 (30)	3 (20)	0.7003	1 (3.3)	1 (2.4)	9 (25.7)	2 (2.8)	**0.0007**
Unknown	1 (5)	1 (6.7)	1	0	4 (9.8)	2 (5.7)	4 (5.6)	1

* *Confirmed by PCR test. (AB): confirmed by positive SARS-CoV-2 antibody test*.

*NED, no evidence of disease; Tx, treatment; SD, standard deviation; m, months; COPD, chronic obstructive pulmonary disease; ILD, interstitial lung disease. Bolded values represented significant results in the p-values*.

### Clinical Features of the Hospitalized Patients

Thirty-five patients required hospitalization. Twenty patients did not require intubation and survived (group A). Among 15 patients in group B, 7 required intubation and 13 were deceased. The intubation rate was 46.7% and only 1 intubated patient survived (14.3%). Two patients died at a second admission, and 1 died in a rehabilitation center after discharge. The case fatality rate based on the hospitalized patients was 37.1 % (13 out of 35) and 12.3 % (13 out of 106) based on all cases.

The tumor and treatment characteristics of those patients in group B are shown in Table 3. The tumor types were breast (*n* = 5), lung (*n* = 3), lymphoma (*n* = 2), GYN (*n* = 2), GI (*n* = 2), and 1 case of unknown primary. Twelve patients received any type of anti-cancer treatment within 3 months, while 6 (40%) received negative impact chemotherapy ≤ 14 days. Most patients had advanced cancer (*n* = 12), while three patients had no evidence of disease (NED). Of note, 13 of the 15 patients in group B had 2 or more comorbidities.

**Table 3:** Clinical characteristics of patients in group B, hospitalized and diseased or requiring intubation.

	**Age**	**Gender**	**2 or more comorbidities**	**Primary tumor**	**Localized tumor or metastatic site**	**Cancer treatment**	**Last treatment to admission day**	**Hospital Days**	**Was intubation required?**	**Outcome**
1	68	Female	✓	Endometrial	Peritoneum	None	N/A	No info		Deceased
2	83	Female	✓	NSCLC	Liver	Dabrafenib*, Trametinib*	N/A	3		Deceased
3	68	Male	✓	Esophageal	Bilateral supraclavicular and lower cervical lymphadenopathy	Cisplatin, Irinotecan	11 days	8	✓	Deceased
4	62	Female	✓	Breast	Chest wall, bone, mediastinal, retroperitoneal LNs	Paclitaxel, Atezolizumab	6 days	12 2^nd^ admission 3 days		Deceased
5	64	Female	✓	Breast	Bone, lung, and pleura	Ibrance, Fulvestrant	28 days	14	✓	Deceased
6	71	Male	✓	Gastric	Stomach	Docetaxel, Oxaliplatin, Leucovorin, 5-FU	14 days	30	✓	Deceased
7	80	Female	✓	Breast	Localized	Paclitaxel	1 day	22 2^nd^ admission 16 days		Deceased
8	65	Male		NSCLC	Mediastinal soft tissue, visceral pleura	Tagrisso, Carboplatin, Alimta	51 days	12		Deceased
9	69	Male	✓	SCLC	LNs, liver, and bone	Carboplatin, Etoposide, Durvalumab	13 days	6		Deceased
10	66	Male	✓	Unknown primary	Lung	Gemcitabine, Carboplatin	51 days	3		Deceased
11	83	Female	✓	Endometrial	NED	Carboplatin, Paclitaxel	79 days	2		Deceased
12	48	Male		Lymphoplasmacytic Lymphoma, WM	Lymph nodes	None	N/A	60	✓	Alive
13	67	Female	✓	DLBCL	NED	None	N/A	23	✓	Deceased
14	77	Female	✓	Breast	Lung/Brain	Adriamycin	10 days	24	✓	Deceased
15	65	Female	✓	Breast	NED	Herceptin*, Letrozole*	N/A	Discharged April 2020	✓	Alive

*NSCLC, non-small cell lung cancer; SCLC, small cell lung cancer; DLBCL, diffuse large B cell lymphoma; WM, Waldenstroms Macroglobulinemia; NED, no evidence of disease*.

At the time of admission, excluding those who were admitted to outside hospitals with incomplete information, 7 (35%) and 11 (73.3%) patients in group A and B had hypoxemia (room air oxygen saturation <92%) (Table 4). Fourteen patients in group A also met other criteria for admission: anemia with or without bleeding from the gastrointestinal tract requiring transfusion, neutropenia with or without fever, mental status changes, or syncope with a burn (Table 4). Three patients in groups A were admitted for other reasons and had incidental findings of positive SARS-CoV-2 infection (Table 4 legend). All admitted patients in group B with available medical records showed respiratory compromise. More than 50% of patients in group A (*n* = 11) required oxygen supplement during the hospital course, and most patients in group B progressed with worsening respiratory status, with 7 requiring mechanical ventilation, all for respiratory failure (Table 4). A number of patients also developed other complications including renal insufficiency, liver function abnormalities, venous or arterial thrombosis and sepsis (Table 4). Lymphopenia, elevation of LDH, and elevation of D-Dimer were very common (Table 4).

**Table 4:** Presentation and admission criteria for admitted patients, as well as their hospital course and complications.

	**Group A *N* = 20 Number/Available** ** (%)**	**Group B *N* = 15 Number/Available** ** (%)**
**Hypoxia at presentation**^**a**^		
Yes	7 (35)	11 (73.3)
No	6 (30)	0 (0)
Unknown	7 (35)	4 (26.6)
**Other criteria for admission at presentation**		
Anemia requiring transfusion	3 (15)	0 (0)
GI bleeding and anemia	2 (10)	0 (0)
Neutropenia ± fever	3 (15)	1 (6.7)
Mental status changes	3 (15)	5 (33.3)
Syncope	1 (5)	0 (0)
Incidental finding^b^	3 (15)	0 (0)
Unavailable information	3 (15)	3 (20)
**Hospital course with progression of hypoxemia**		
Unknown	6 (30)	2 (13.2)
Room air only	3 (15)	0 (0)
Oxygen supplement by:		
NC or Ventruri mask	8 (40)	2 (13.2)
HFNC	2 (10)	0 (0)
100% NRB	1 (5)	4 (26.6)
Ventilator	0 (0)	7 (46.6)
Prophylactic intubation	0 (0)	0 (0)
Intubation due to respiratory failure	0 (0)	7 (100)
**Hospital course with other complications**^**c**^		
Renal insufficiency^d^	10/14 (71.4)	7/10 (70)
LFT elevations	10/14 (71.4)	10/10 (100)
DVT or PE	1 (5)	0 (0)
A-Fib or MI	1 (5)	2 (13.2)
Bacterial pneumonia, bacteremia	1 (5)	3 (20)
**Other lab abnormalities**^**c**^		
Lymphopenia	13/14 (92.9)	10/10 (100)
Elevation of LDH	11/12 (91.7)	10/10 (100)
Elevation of D-Dimer	11/12 (91.7)	9/9 (100)

a *Hypoxia is defined as oxygen saturation at room air to be <92%*.

b *The reasons of admission for the 3 patients who had incidental findings of SARS-CoV-2 infection status were: social admission (n = 1), displaced nephrostomy tube (n = 1), and large neck mass requiring emergency tracheostomy (n = 1)*.

c *The percentages in those categories are calculated based on the number of patients with available information, not the total number of patients in the respective groups*.

d *Abnormal lab values: any value outside the upper or lower limit of normal reference value per hospital lab*.

*GI, gastrointestinal; NC, nasal cannula; HFNC, High flow nasal cannula; NRB, non-rebreather; LFT, liver function tests; DVT, deep vein thrombosis; PE, pulmonary embolism; A-Fib, atrial fibrillation; MI, myocardial infarction; LDH, Lactate dehydrogenase*.

In comparing the clinical characteristics to differentiate patients in group A from group B, there were no statistical differences in age, active cancer status, treatment status, number of comorbidities, whether chemotherapy with negative impact had been given or not, or given within 30 days (Table 2). There was no statistical difference between group A and B in terms of the duration of any treatment or the duration of treatment with negative impact agents prior to the diagnosis of SARS-CoV-2 infection (Table 2). The difference in the percentage of 2 or more comorbidities between group A and B was not statistically significant (Table 2). However, hypoxemia at presentation was significantly more common in group B than in group A (*p* = 0.0271), suggesting that a respiratory compromise at presentation was an important adverse predictive factor for poor survival.

**Figure 2:**
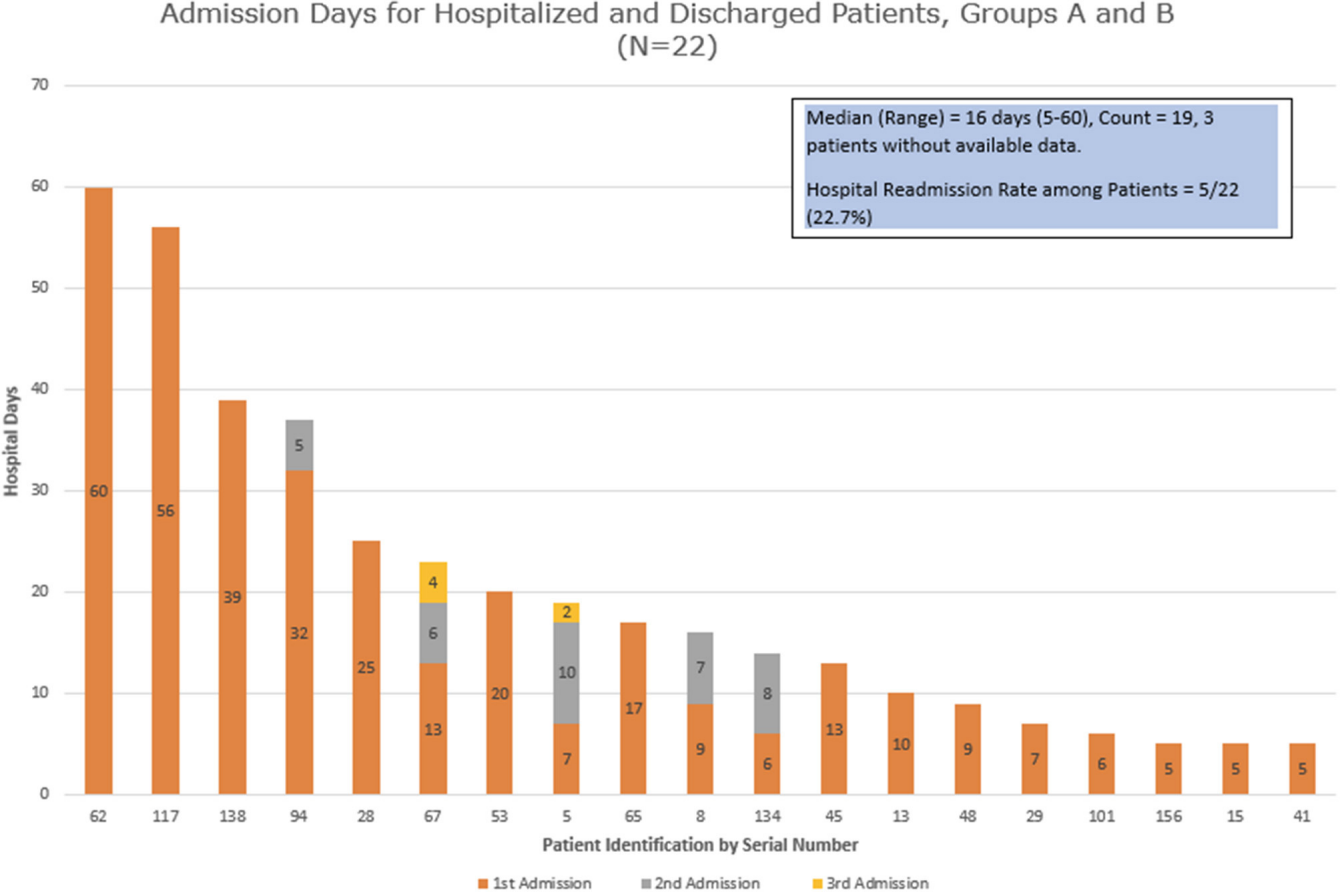
Admission days for hospitalized and discharged patients.

We examined the hospital admission days for all the patients who were admitted and discharged (group A + 2 patients from group B) (Figure 2). The median duration of days for their first admission was 10, ranging between 5 and 60 days. Five patients (27.8%) had a second admission, while 2 of the 5 patients then had a third admission. The next admission dates were 1–27 days from the last discharge, with a median of 5 days. The median duration of cumulative hospitalized days (including all admissions) was 16 with a range of 5–60 days.

### Characteristics of Patients Under Home Isolation

The right side of Table 2 shows the clinical characteristics of patients in groups C and D. There were 30 patients in group C (6 due to suspicious surveillance CT scan, 3 due to history of close exposure and 21 due to retrospective identification with positive COVID-19 antibody test). Those patients did not show detectable symptoms during subsequent follow up or retrospective recall. The months of diagnosis was not able to be ascertained in 18 patients, as they were captured by positive COVID-19 antibody tests, but could not recall any previous symptoms or close contact. Group C was noted to have the numerically lowest percentage of patients having presence of disease (46.7%), and the numerically highest percentage of patients in the category of NED + treatment (40%). Group D had 41 patients who had various degrees and constellations of COVID-19 symptoms with subsequent recovery. Although 76.7% and 70.7% patients in group C and D were receiving active treatments, only 20% and 26.8% were receiving myelosuppressive treatments, respectively, and an additional 10 and 4.9% were receiving immunomodulating treatments, respectively; the rest of the patients were receiving endocrine therapy. Although there was a numerical increase in patients taking negative impact treatment in group C vs. group D, it was not statistically significant (*p* = 0.8002).

### Comparison of Clinical Factors Between the Hospitalized Patients and At-Home Recovery Patients

We then compared the clinical characteristics of patients who were hospitalized (groups A + B) to patients who recovered at home (groups C + D) (Table 2). Patients in Groups A + B were older patients (mean age 71 vs. 63 years old, *p* = 0.000299). There was no difference between groups A + B vs. groups C + D regarding the factors of having active disease, receiving anti-cancer treatment, or both. Receiving negative impact therapies was a distinguishing factor (62.9 vs. 32.4%, *p* = 0.0036), which was driven mainly by receiving myelosuppressive treatment (45.7 vs. 23.9%, *p* = 0.0275), while receiving immunosuppressive or immunomodulating treatments did not show difference between groups A + B vs. groups C + D (Table 2). Patients taking negative impact treatment within 30 days of admission was 48.6% in groups A + B and 36.6% in groups C + D, which was also statistically significant (*p* = 0.017). Groups A + B have significantly more patients harboring 2 or more other comorbidities (74.3 vs. 47.9%, *p* = 0.0124).

Patients in group C + D had a longer median duration on treatment (11.2 months) than that in group A + B (3.0 months), which was statistically significant, although opposite to the intuitive prediction. Another way of examination by mean + standard deviation (SD) showed similar trend and same *p*-value. The duration of negative impact treatments between Group A + B vs. group C + D did not show statistical significance.

At a baseline assessment of the performance status (PS) of the patients, patients in groups A + B had more patients with PS of 2 or above than groups C + D, indicating patients who needed hospital care had poorer performance status at baseline. The baseline immune status, taking into account of absolute neutrophil counts, or absolute lymphocyte counts or albumin levels, was similar in all groups.

A multivariate analysis was carried out to explore the most important risk factors that were associated with a more severe form of SARS-CoV-2 infection with hospitalization (group A + B) vs. home recovery (group C + D) (Table 5), we observed that older age (Odds Ratio, OR = 1.01, 95% CI: 1.00–1.02) (*p* = 0.004) was significant. According to this statistical model, OR>1 indicates more likelihood for hospitalization, and the odds increases by 1% for each additional year of age. Being on active myelosuppressive treatment (OR = 0.78, 95% CI: 0.66–0.93) (*p* = 0.006) and having a poorer performance status ≥ 2 vs. PS 0–1 (OR = 0.64, 95% CI: 0.49–0.84) (*p* = 0.002) were significantly associated with a more severe status of infection. Negative impact treatment as a whole, and 2 or more comorbidities, although significant predictors in univariate analysis, were not significant in multivariate analysis.

**Table 5:** Multivariate logistic regression analysis on risk factors associated with a more severe status of SARS-CoV-2 infection: group A + B (hospitalized patients) vs. group C + D (home recovery patients).

**Characteristics**	**Univariate**		**Multivariate**	
	**OR (95% CI)**	***P*-value**	**OR (95% CI)**	***P*-value**
Age	1.06 (1.03–1.11)	0.001	**1.01 (1.00**–**1.02)**	**0.004**
Gender:				
Male (vs. Female)	1.96 (0.82–4.67)	0.125		
Cancer types:				
Hematological (vs. Solid tumors)	2.73 (0.76–10.17)	0.119		
Disease status:				
Presence of disease (vs. NED)	1.86 (0.82–4.41)	0.146		
Treatment and disease:				
NED + Tx (vs. NED no Tx)	0.73 (0.19–2.93)	0.701		
Disease no Tx (vs. NED no Tx)	1.37 (0.26–7.02)	0.647		
Disease + Tx (vs. NED no Tx)	1.57 (0.50–5.60)	0.457		
Treatment:				
No treatment (vs. treatment)	0.95 (0.36–2.34)	0.909		
Treatment: (No vs. Yes)				
Myelosuppressive	0.37 (0.16–0.88)	0.025	**0.78 (0.66**–**0.93)**	**0.006**
Immunosuppressive	0.92 (0.41–2.09)	0.842		
Immunomodulating	0.45 (0.12–1.75)	0.239		
Any above negative impact	0.28 (0.12–0.65)	0.004		
Negative Impact <30 days	0.35 (0.15–0.80)	0.014		
Time duration of active treatment (m)	0.98 (0.94–1.00)	0.138		
Time duration of tx with negative impact (m)	0.98 (0.93–1.01)	0.298		
Comorbidities:				
2 or more factors (vs. 0 or 1 factor)	3.14 (1.33–7.98)	0.012		
HTN (No vs. Yes)	0.43 (0.16–1.04)	0.072		
DM (No vs. Yes)	0.71 (0.30–1.69)	0.433		
HLD (No vs. Yes)	0.39 (0.16–0.90)	0.028		
h/o PE/DVT (No vs. Yes)	0.36 (0.08–1.44)	0.146		
COPD/ILD/asthma (No vs. Yes)	0.89 (0.31–2.79)	0.828		
Baseline lab for immune status:				
Normal (vs. abnormal)	0.56 (0.22–1.44)	0.226		
Unknown (vs. abnormal)	0.25 (0.01–1.89)	0.241		
Performance				
PS ≥2 (vs. PS 0–1)	12.19 (2.89–83.82)	0.002	**1.57 (1.19–2.06)**	**0.002**

*Bolded values represented significant results in the p-values*.

### Time Kinetics of Persistent SARS-CoV-2 Virus Status

**Figure 3:**
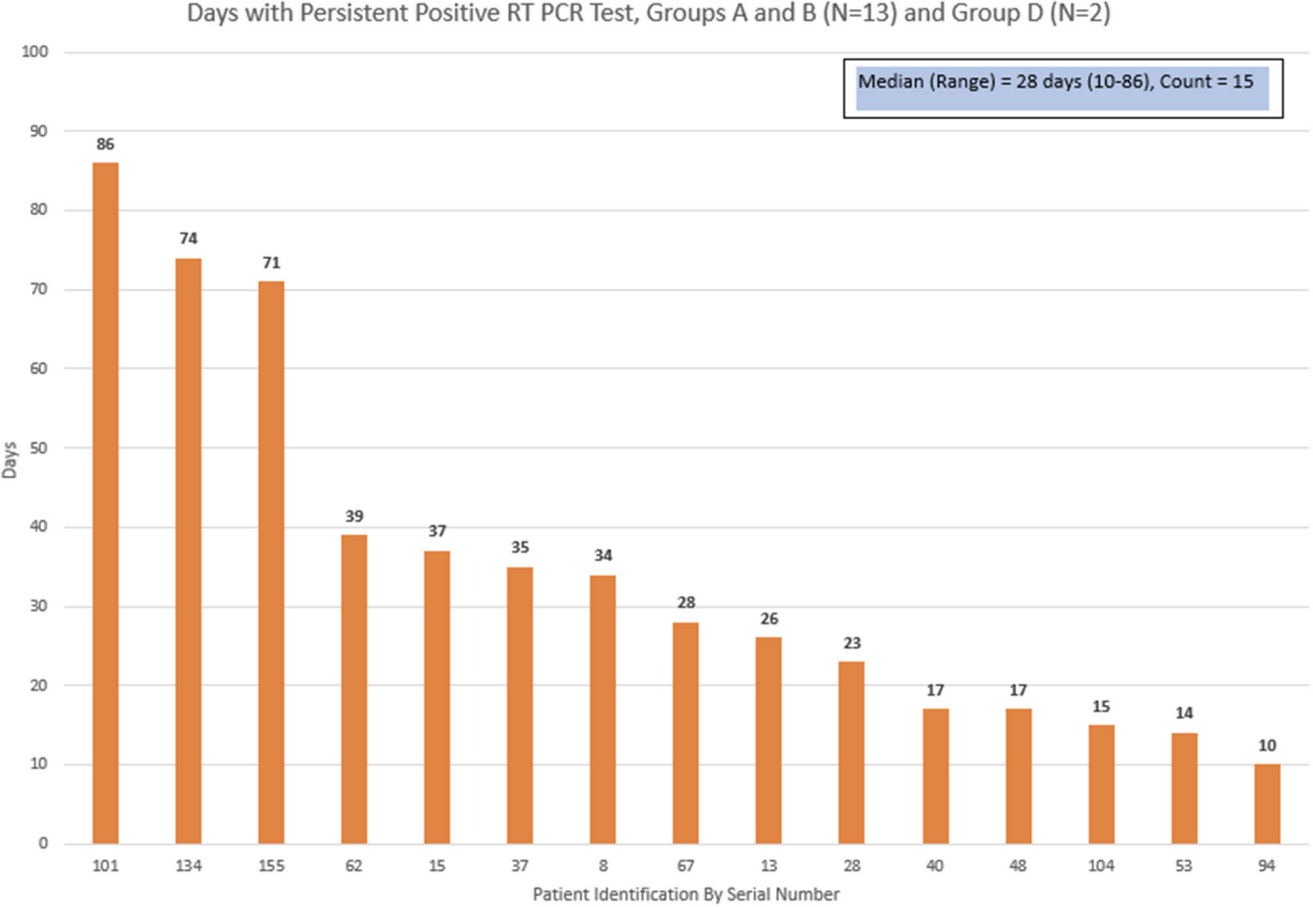
Time kinetics in persistent positivity of COVID PCR test.

Thirteen patients who were discharged home from groups A + B, and 2 patients from group D had a subsequent repeat positive COVID-19 RT-PCR test. The days between the initial positive test and the last positive test was defined as “positive duration.” The median duration for the positive duration was 28 days, with a range of 10–86 days (Figure 3).

### Re-initiation of Cancer Treatment

The details of patients receiving treatment prior, during and after their SARS-CoV-2 diagnosis are shown in Table 6. Among the 15 patients in group A + B who survived hospital admission and were receiving treatment prior to SARS-CoV-2 infection and the 2 patients waiting to start new treatment, 9 (52.9%) patients started treatment after recovery, with a median delay of 40 days (range 14–75 days). In group C, 22 patients were receiving treatment and 2 were waiting to start treatment, and all of them continued or started treatment as planned during their presumable SARS-CoV-2 infection duration. Two patients were supposed to start adjuvant chemotherapy after surgery, and they had a delayed start. The on-schedule rate was 92.3% (24/26). In group D, 29 patients were on treatment prior to SARS-CoV-2 diagnosis, and 15 continued on schedule, while 13 restarted after a delay, with an on-schedule rate of 51.7%, and continuation rate of 96.6%. The median duration of delay was 17 days (range 6–31) based on cohort 1 group D data only, as the recall data for the duration of symptoms from cohort 2 group D patients was considered inaccurate.

**Table 6:** Treatment before, during, and re-initiation after SARS-CoV-2 diagnosis.

	**Group A + B**	**Group C**	**Group D**
Total patients	35	30	41
Total on treatdment before COVID-19 pandemic	15 (42.9)	22 (73.3)	29 (70.7)
Myelosuppressive	8 (22.9)	5 (16.7)	11 (26.8)
Immunosuppressive	2 (5.7)	0	1 (2.4)
Immunomodulating	2 (5.7)	3 (10)	2 (4.9)
Non-myelosuppressive or endocrine or radiation	3 (8.6)	14 (46.7)	15 (36.6)
Waiting to start treatment before or during COVID-19 pandemic	2 (5.7)	2 (6.7)	3 (7.3)
Treatment during pandemic and SARS-CoV-2	0	24 (80)	15 (36.6)
Diagnosis			
On schedule	0	22 (73.3)	15 (36.6)
New start	0	2 (6.7)	0
Myelosuppressive	0	7 (23.3)	4 (9.8)
Immunosuppressive	0	0	0
Immunomodulating	0	3 (10)	0
Non-myelosuppressive or endocrine or radiation	0	14 (46.7)	11 (26.8)
Delayed: Re-initiation after SARS-CoV-2 diagnosis	9 (25.7)	0	13 (31.7)
Or start new treatment	0	2 (6.7)	3 (7.3)
Myelosuppressive	7 (20)	2 (6.7)	6 (14.6)
Immunosuppressive	0	0	1 (2.4)
Immunomodulating	1 (2.9)	0	2 (4.9)
Non-myelosuppressive or endocrine or radiation	1 (2.9)	0	7 (17.1)
Duration of delay (from discharge to restart):			
Median (range)	40 (14–75)	57.5 (37–78)	17 (6–31)^*^
Restart rate (%)	52.9 (9/17)	N/A	N/A
On-schedule rate (%)	0	92.3 (24/26)	51.7 (15/29)
Continuation rate (%)	n/a	100 (26/26)	96.6 (28/29)

* *Based on cohort 1 data only*.

## Discussion

We report results from a registry study at a community cancer center located in New York City, the epicenter for COVID-19 pandemic in the United States. Among 106 patients who had a confirmed SARS-CoV-2 diagnosis, 33.0% of the patients required hospitalization and the case fatality rate was 37.1% among those hospitalized and 12.3% for the entire cohort. Among the patients who required hospitalization, not having hypoxemia at presentation appeared to be a significant factor for survival. Other than that, we could not identify distinguishing factors such as age, comorbidity, tumor characteristics, or treatment characteristics to predict mortality. However, we did identify multiple factors that were associated with a more severe status of SARS-CoV-2 infection for those who required hospitalization vs. those who recovered at home. These factors were: (1) older age, (2) use of treatments with potential negative impact to immune system, which was represented mainly by patients receiving myelosuppressive therapies, (3) having more than 2 comorbidities, and (4) a baseline poor performance status. Among them, older age, having myelosuppressive chemotherapy, and a baseline poor performance status emerged in multivariate analysis as strong, significant risk factors for a severe form of SARS-CoV-2 infection.

The relationship of cancer and outcome from SARS-CoV-2 infection has been the topic of multiple studies (2, 5, 7–11). The vulnerability of cancer patients to severe SARS-CoV-2 complications and increased mortality is presumably owing to immunosuppression from either the presence of disease and/or the detrimental effects from anti-cancer treatment. The tumor burden may presumably induce secondary decline in metabolism, nutritional status, and even more immunosuppression, which is not well-defined. In that regard, the study from Dai et al. suggested that patients with metastatic disease had the highest frequency of severe events, where the outcome was similar in patients without metastatic disease to patients without cancer history (12). However, multiple published studies generated conflicting results of whether chemotherapy alone stands as a sole negative factor for the severity of the infection. While multicenter studies from China suggested detrimental effects from anti-cancer treatment (2, 12), studies from the COVID-19 and Cancer Consortium (CCC) and MSKCC did not support it (9, 10). Even in multiple myeloma patients, the study from Mount Sinai revealed no bearing of treatment drug exposure (13).

Our observations provide evidence to support the above presumptions and show that treatment with negative impacts therapies resulted in worse outcomes. Importantly, myelosuppressive chemotherapy was clearly a differentiating factor, while immunomudulating therapies (namely immune check-point inhibitors) were not. Our study was unique in multiple ways. We focused on chemotherapies with negative impact instead of on all anti-cancer treatment, as a large portion of these treatments are of endocrine or non-myelosuppressive nature which may not negatively affect a patient's immune defense. While most of the previous studies analyzed data on symptomatic hospitalized patients, we selected our control group to include patients who recovered at home and asymptomatic (group C), or mildly to moderately symptomatic (group D). We demonstrated that treatments with negative impact resulted in worse symptoms leading to hospital care but did not differentiate outcomes leading to death or survival. Age and comorbidity in cancer patients fared as unfavorable factors as well, as depicted in all other published studies for both cancer patients and for the general population. Our study did not show a relationship of metastatic disease with worse outcomes. Performance status is a way to quantify the general well-being and activities of daily living in cancer patients. This measure has been widely used in determination of a patient's eligibility for aggressive chemotherapy. Interestingly, patients in groups A + B had a higher percentage of decreased performance status, suggesting “compromised functional status” as a predictive marker for severe infection as well. Patients in group C + D had a statistically significant longer median duration on treatment (11.2 months) than that in group A + B (3.0 months). Although this observation may be opposite to the intuitive prediction, one could attempt a presumptive hypothesis that the patients in group C + D had undergone a natural selection to be those who had preserved PS to continue treatment for a prolonged time.

Based on the findings in our study, we would recommend consideration of decreasing the exposure to treatments with negative impact on patients who are older, or with compromised functional status and have comorbidities during the height of the COVID-19 pandemic. To address the concerns mentioned above, the medical oncology community has already developed guidelines for treatment modifications and adopted practice-changing strategies. Most of them considered individualized assessment incorporating both disease and treatment factors (14).

The case fatality rate (CFR) in our study was 37.1% among hospitalized patients and 12.3% among all the study population. This is comparable to other studies conducted at New York City, most notably the study from Montefiore Hospital system, which reported a CFR of 28% among mainly hospitalized cancer patients (5). Memorial Sloan Kettering Cancer Center reported CFR of 12% in overall symptomatic cancer patients; considering a reported 40% admission rate, the CFR would be 30.1% among hospitalized/admitted patients (9). Socioeconomic status, racial disparity, timely pursuit of medical care, and access to critical care resources in an overwhelmed community hospital may contribute to the poor outcome of our hospitalized patients, in addition to their medical conditions. On the other hand, the CFR in the general population in the New York City during the same period was 5.2% and 6% from 2 reports (5, 9), much lower than that of the cancer patients. Furthermore, our cancer patients who contracted SARS-CoV-2 infection and required hospital admission had a median cumulative admission of 16 days, while the New York City average was 3.9 days (15). In addition, they required a longer time for virus clearance (median of 28 days), while it was 4–17 days in general population (16, 17). As we defined positive virus interval to be between 2 positive RT-PCR results, this becomes an underestimation of the true virus clearance time. Other studies also reported similar observation of prolonged virus clearance (13, 18). The above results collectively confirmed that cancer patients had a higher risk for more severe events when contracted with SARS-CoV-2 infection.

In this study, we reported for the first time that cancer patients with SARS-CoV-2 infection might exhibit no symptoms. They not only appeared “healthy,” but also actually remained healthy, as many of them were able to receive planned anti-cancer treatment on schedule. This group (group C) is comprised of 28.3% (30/106) of the all patients, with 46.6% of them having presence of tumor and 76.7% of them taking treatment. Likewise, patients with similar characteristics might have mild symptoms who were able to recover at home and almost all resumed planned treatment. It is interesting to note that the asymptomatic carrier rate of 28.3% in cancer patients was moderately lower than the 40% in the public that was reported by the Center of Disease Control (CDC) in July 2020 (19).

We also attempted to give assessment of the patients' immune status at baseline. The function of immune system may be measured by both humoral and cellular immunity. Judging from the routine clinical labs, the immune status may be partially measured by the absolute neutrophil count (ANC) and the absolute lymphocyte count (ALC). The quantitative immunoglobin level is a more direct assessment of the humoral immunity, but it is not routinely tested. With a composite assessment of ANC, ALC, and albumin values taken at a visit prior to COVID-19 pandemic, we noted that about 60–70% patients had all normal labs, and no difference was found among different groups, or between groups A + B vs. C + D. These results suggest that not all patients with cancer and/or on treatment are rendered a status of severe immunosuppression to the point of being unable to fight the coronavirus. In fact, in our previous study on the generation of protective neutralizing antibodies after H1N1 vaccination in 2015, cancer patients' response to vaccination was as good as the healthy controls (20). Similarly, in the patient cohort of multiple myeloma at Mount Sinai hospital, a majority of the patients also mounted anti-COVID-19 antibodies (13). Furthermore, 52.9% of patients (groups A + B) who required hospital admission and 96.6% of patients who had mild symptoms (group D) were able to start or restart anti-cancer treatment after a hiatus. This observation demonstrated the proportion and degree of complete recovery of cancer patients from SARS-CoV-2 infection, albeit with a longer time course.

The strength of this study is the inclusion and analysis of the characteristics of asymptomatic patients. Some of those rose to attention due to suspicious CT findings or a history of close exposure; and a large portion was discovered with antibody screening in late May and June. This part of the study cohort is not covered in most of the published studies (2, 5, 8, 9, 13). This research methodology enabled us to have a glimpse of the base of the pyramid, to those who had cancer and mild symptoms and to examine their cancer burden and treatment status. The inclusion of cohort 2, with consecutive patients identified by electronic medical records for positive COVID-19 antibody tests, also significantly reduced a selection bias of not encompassing all patients who recovered at home with no or mild symptoms. Another strength is the analysis of patients' baseline immune status and performance status prior to the pandemic and the identification of a baseline performance status as a predictive marker for the severity of disease.

There are multiple limitations in our study. First, our data collection reflected a relatively small sample size. Second, about 69.8% of patients in this study were females, and breast cancer was the most prevalent diagnosis. Very small numbers of hematological malignancies were represented here, therefore raising cautions in generalizing the conclusion to patients with other malignancies. Third, we also excluded patients who were admitted to outside hospitals who lacked confirmatory information. Lastly, we excluded patients with suspicious clinical findings but negative or unconfirmed COVID-19 antibody test results. It is possible that some patients with true infection did not develop antibodies to COVID-19, or the presence of antibodies in some asymptomatic and mildly symptomatic patients was transient and diminished at the time of the test. In a recent publication, 40% of asymptomatic patients vs. 12.9% of symptomatic patients became seronegative in the early convalescent phase (21).

Overall, our observations should add valuable information to the rapidly accumulating world evidence of cancer and SARS-CoV-2 infection. Systemic anti-cancer treatment with a potential of negative impact to the immune system, particularly myelosuppressive chemotherapy, advanced age, with compromised functional status, and having more than 2 comorbidities were unfavorable factors associated with more severe infection status and hospital admissions but not for in-hospital mortality. Cancer patients not only have a higher mortality rate than the general population, but they also have longer hospital admission stay and protracted virus clearance time. On the other hand, patients with cancer on active treatment still may have mild disease, improve without hospitalization, and re-initiate anti-cancer treatment after recovery from SARS-CoV-2 infection. Proactive mitigation of modifiable factors and a careful balance of benefits and risks associated with anti-cancer treatment is warranted to safely navigate our cancer patients' course during the COVID-19 pandemic.

## Data Availability Statement

The raw data supporting the conclusions of this article will be made available by the authors, without undue reservation.

## Ethics Statement

The studies involving human participants were reviewed and approved by The Maimonides Medical Center Institutional Review Board (IRB # 2020-04-05). Written informed consent for participation was not required for this study in accordance with the national legislation and the institutional requirements.

## Author Contributions

DL, NS, BD, and YX: conception of research. KB, SB-R, JD'S, YH, JL, TM, LM, PM, PR, BD, and YX: provision of patient information. DL, YW, ST, VN, BD, and YX: data collection and analysis. YW: statistical analysis. DL, YW, BD, and YX: principal manuscript writing. All authors contributed to the manuscript writing and approval.

## Conflict of Interest

The authors declare that the research was conducted in the absence of any commercial or financial relationships that could be construed as a potential conflict of interest.
